# Mrs. Evelyn Rachel Bates

**Published:** 1933

**Authors:** 


### MRS. EVELYN RACHEL BATES, M.R.C.S., L.R.C.P.

ANOTHER younger member of the Society passed away, deeply mourned, on 18th February. Mrs. Bates was the younger daughter of the late Lionel Edward Pyke, Q.C., of Kensington. Educated at Priorsfield School, Miss Pyke went up to Newnham

College, Cambridge, in 1916, where she read for the Natural Sciences Tripos and the M.B. examinations. At school she had been an excellent lacrosse player, and she played continuously for Cambridge while she was up. She was captain of the Cambridge team during her last year and led them to victory against Oxford. Shortly before the Tripos she developed appendicitis, and in consequence of this interruption of residence did not keep the terms necessary for a Cambridge degree. A Cambridge contemporary writes of her : "I always thought her a person of quite remarkable courage and capacity for clear thought. One always learnt from her, too, which were the books, the plays and the music worth attending to," for Mrs. Bates was a more than average violinist and played in orchestras at Cambridge and in London.

On leaving Cambridge she continued her medical studies at the London Hospital, and took the double qualification M.R.C.S., L.R.C.P., in 1924. She then worked for two years as a Research Assistant in the Medical Unit at the London Hospital under Professor Ellis. "The problems on which she was engaged," he says in a letter, "were the significance of the occurrence of yeasts of the Monilia type in the mouth and intestinal tract of patients with pernicious anæmia and, secondly, an attempt to determine if the Dick reaction was affected in patients with acute nephritis. The results of these investigations were never published, but were of very considerable interest." After leaving the Medical Unit Laboratory in 1926 Miss Pyke was Pathological Assistant in the Bernhard Baron Institute of Pathology, and also worked in the Hale Clinical Laboratory.

In February, 1929, she married Mr. R. M. Bates, F.R.C.S., and accompanied him to Bristol on his appointment as Resident Medical Officer at Stoke Park Colony. At the time of his appointment it was not known that Mrs. Bates had a medical qualification, but shortly after her arrival her laboratory experience and pathological knowledge were so constantly in demand that she was appointed Clinical Pathologist to the Colony. Her death from pneumococcal meningitis following influenza came as a great shock to her friends and colleagues. Although Mrs. Bates had only recently come to Bristol, she had impressed everyone who met her by her delightful personality and many-sided ability.

